# Crushing Mechanics and Flour Properties of Wheat Under Different Graded Crushing Durations in a Blade Crusher

**DOI:** 10.3390/foods15162935

**Published:** 2026-08-21

**Authors:** Chi Zhang, Jiyun Hu, Qin Xu, Haihong Zhang, Rangling Li

**Affiliations:** 1 School of Mechanical and Electrical Engineering, Henan University of Technology, No. 100 Lianhua Street, Zhengzhou 450001, China; zhangchi610123@163.com (C.Z.); xuqin@haut.edu.cn (Q.X.); fluent@haut.edu.cn (H.Z.); lirangling@haut.edu.cn (R.L.); 2 School of Rail Transit, Henan Mechanical and Electrical Vocational College, No. 1 Taishan Road, Zhengzhou 451191, China

**Keywords:** graded crushing, crushing mechanics, mechanical models, particle size distribution

## Abstract

This study investigates the effects of different graded crushing durations in a blade crusher on the crushing mechanics of wheat and the properties of the resulting flour. Mechanical models were established for blade–particle collisions, radial sliding of particles along the blade surface, and particle–chamber wall collisions. Under reasonable simplifying assumptions, the models analytically characterize the theoretical relationships of impact force and crushing energy with blade rotational speed, rotational radius, and particle incidence angle. The models were used to provide a qualitative mechanistic interpretation of the experimental trends rather than to quantitatively predict flour particle size distribution or damaged starch content. Two graded crushing processes were evaluated, with crushing durations of 10 s per pass (F10) and 15 s per pass (F15). Observation of particle-size evolution during the crushing of wheat particles showed that as the number of crushing passes increased, the proportion of coarse particles continuously decreased, the proportion of fine particles gradually increased, and the proportion of intermediate-sized particles initially increased and then decreased, demonstrating a progressive coarse-to-fine fragmentation pattern. Particle size analysis of the resulting wheat flour showed that the particle size distribution for the F15 process peaked below 5 μm and shifted toward smaller particle sizes relative to that for the F10 process. Nevertheless, the wheat flour obtained from both processes exhibited relatively concentrated particle size distributions, with Span values ranging from 2.46 to 2.68. Damaged starch content increased significantly with the number of crushing passes and was generally higher for the F15 process than for the F10 process. Moisture content decreased from 14.30% to 12.86% under the F10 process and from 14.25% to 12.73% under the F15 process, whereas ash content ultimately increased to 0.48% under both processes. Protein content initially increased and subsequently decreased under both processes. These findings provide experimental evidence for the effects of graded milling on grain refinement, starch damage, and physicochemical composition of wheat flour.

## 1. Introduction

Wheat is one of the most important cereal crops worldwide and serves as a primary source of carbohydrates and proteins for human nutrition [1]. Wheat flour is the principal intermediate product for a wide range of cereal-based foods, including bread, noodles, biscuits, and steamed products. Milling is therefore a fundamental process in the wheat industry, with the primary objective of achieving the controlled separation and size reduction of wheat kernels to obtain flour fractions suitable for different food applications. Traditionally, wheat flour is produced through multi-stage roller milling systems. In recent years, however, high-speed impact milling equipment, such as hammer and blade mills, has attracted increasing attention because of its compact structure, flexible operation, high crushing efficiency, and potential applications in whole-wheat flour production, specialty flour processing, and grain pretreatment [2,3].

Particle comminution during milling is far more than a simple reduction in particle size. Fundamentally, it is a mechanochemical process in which external mechanical energy is transferred to wheat kernels through impact, compression, shear, and friction, resulting in crack initiation, crack propagation, and ultimately particle fragmentation. The magnitude and distribution of impact forces, together with the cumulative mechanical energy input, determine the breakage behavior of wheat particles and directly influence particle size distribution, damaged starch formation, and flour composition [4,5,6]. From the perspective of food engineering, wheat milling represents a typical “process–structure–property” coupling, in which mechanical treatment modifies the endosperm structure and particle characteristics, thereby affecting the physicochemical properties, dough behavior, and end-use quality of wheat flour [7,8]. Consequently, understanding the mechanisms governing particle fragmentation is essential not only for improving milling efficiency but also for regulating flour functionality.

Among the quality attributes of wheat flour, particle size distribution is regarded as one of the most important structural parameters because it links the milling process with flour composition and product quality [9,10]. Numerous studies have demonstrated that flour particle size significantly affects damaged starch content, water absorption, hydration behavior, rheological properties, gelatinization characteristics, and the quality of final wheat-based products [11,12]. Therefore, controlling particle-size evolution during milling has become an important research topic in cereal science.

Considerable progress has been made in understanding wheat milling from both the mechanical and processing perspectives. Previous studies have investigated the mechanical properties of individual wheat kernels under compression or shear loading, including elastic modulus, fracture strength, and failure behavior under different moisture conditions. Other studies have focused primarily on optimizing the operating parameters of roller mills—such as roll gap, roll speed, and milling flow—to improve flour yield and quality [13,14,15]. Nevertheless, several important scientific questions remain unresolved. First, for high-speed blade crushing, most existing studies have mainly established empirical relationships between operating parameters and final flour characteristics, whereas the transient collision behavior, particle fragmentation mechanism, and energy transfer during repeated impacts remain insufficiently understood [16,17]. Second, most investigations have concentrated on the properties of the final flour, while the dynamic evolution of particle size distribution during successive crushing passes has rarely been systematically characterized [18,19]. Consequently, the progressive transformation of coarse particles into intermediate and fine particles throughout the entire graded crushing process remains poorly understood.

Unlike previous studies that have mainly focused on the properties of final milling products, the present study focuses on the pass-by-pass evolution of wheat particles during graded crushing in a high-speed blade crusher. The novelty of this work lies in characterizing the progressive transformation of particle-size fractions through successive crushing–sieving cycles under different crushing durations per pass and relating the observed changes to damaged starch and flour physicochemical properties. In addition, analytical descriptions of blade–particle impact, radial particle motion along the blade surface, and particle–chamber-wall collision are incorporated to provide a mechanical interpretation of the observed fragmentation behavior.

## 2. Materials and Methods

### 2.1. Experimental Materials

The experimental material selected was Zhengmai 9023, a high-gluten wheat variety widely cultivated in Henan Province, with a crude protein content of 14.9% and a bulk density of 804 g/L. Samples totaling 2 kg were obtained from the experimental fields of Henan University of Technology. After natural drying and the removal of impurities, the samples were stored at 4 °C for later use. Prior to experimentation, the cleaned wheat samples were brought to room temperature and sequentially sieved through Taylor sieves. Of these, 4% were large grains (retained on the Taylor 6-mesh sieve with a pore size of 2.8 mm), 95% were medium grains (retained on the Taylor 8-mesh sieve with a pore size of 2.5 mm), and 1% were small grains (collected in a tray). Before crushing, the large- and medium-sized wheat kernels were conditioned to a moisture content of 15% at room temperature (20–25 °C) and allowed to equilibrate for 24 h before use.

### 2.2. Experimental Equipment

The 400Y blade mill is produced by Yongkang Bo Ou Hardware Products Co., Ltd., Jinhua, Zhejiang Province, China. The working parameters are shown in Table 1. The NKT2020-L laser particle size analyzer is produced by Shandong Nike Analytical Instrument Co., Ltd., Jinan, Shandong Province, China. SDmatic Damaged Starch Analyzer from Chopin Technologies, Paris, France. The ML4002 Electronic Balance (Sensitivity 0.01 g) is produced by Mettler Toledo, Zurich, Switzerland. The Test Sieve is produced by Shaoxing Shangyu Instrument Equipment Co., Ltd., Shaoxing, Zhejiang Province, China. The SYS-21J Vibrating Screen is produced by Quanzhou Huadun Machinery and Equipment Co., Ltd., Quanzhou, Fujian Province, China. The working parameters are shown in Table 1.

### 2.3. Graded Crushing Technology Flow

**Figure 1:**
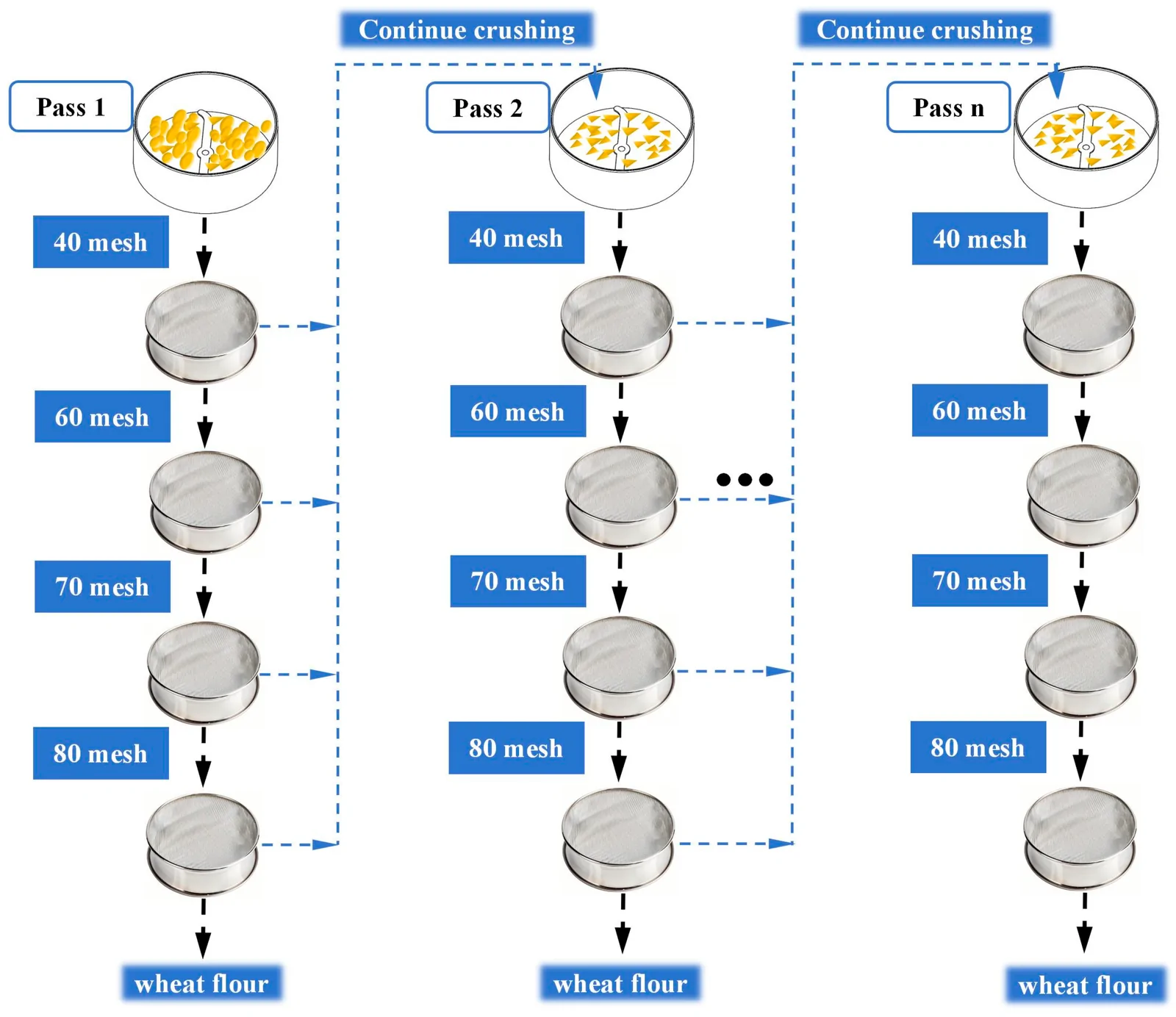
Graded preparation process diagram.

Take 1 kg wheat sample for graded crushing. The sample was crushed in 5 batches (0.2 kg per batch) in sequence. After crushing, the material is sieved through standard experimental sieves of 40 mesh, 60 mesh, 70 mesh, and 80 mesh in sequence. The material passing through the 80-mesh sieve is collected and defined as wheat flour, denoted as the first-pass flour. Subsequently, the sieved materials from each layer are combined and crushed again, and the above sieving process is repeated. The material passing through the 80-mesh sieve is denoted as the second-pass flour. This operation is repeated, with the oversized material being crushed and sieved each time, and the pass number is recorded in sequence, until the cumulative mass of material passing through the 80-mesh sieve exceeds 80% of the total sample mass. Two crushing durations—10 and 15 s per pass—were used, and the corresponding treatments were designated F10 and F15, respectively. These crushing durations were selected based on preliminary trials. After 10 s of crushing, the mass fraction of intact wheat kernels was below 2% of the initial sample mass, indicating that most kernels had undergone initial breakage. Extending the crushing duration to 15 s further reduced the mass fraction of intact kernels to below 1.5%. Thus, 10 and 15 s were selected to represent two different crushing durations per pass while ensuring effective initial breakage of the wheat kernels. The graded preparation process is shown in Figure 1.

The quality of wheat grains was recorded before and after each crushing pass, as well as after screening, to monitor material loss. In the F10 and F15 processes, the quality loss during the entire classification crushing and screening process is 1.1% and 1.3%, respectively. The loss of materials is mainly attributed to the adhesion of particles to the walls of the crushing chamber and screen surface, as well as the fine dust generated during the screening process.

### 2.4. Particle Size Analysis

Wheat flour particle size was analyzed on an NKT2020-L laser particle size analyzer using air as the dispersant. The instrument settings included a particle refractive index of 1.52 and an absorption index of 0.1. The measurement range was 0.1~800 μm, with the obscuration maintained at 10~15% during operation. This study used a quantity–particle size distribution for data statistics. All samples were measured three times, and results are presented as averages.

### 2.5. Determination of Damaged Starch Content in Wheat Flour

According to GB/T 31577-2015 [20] “Grain and Oil Inspection-Determination of Damaged Starch in Wheat Flour-Amperometric Method”, the damaged starch content of wheat flour was measured using a Chopin Damaged Starch Analyzer, Chopin Technologies, Paris, France.

### 2.6. Determination of Basic Physical and Chemical Indicators of Wheat Flour

Refer to AACC Method 44-19 for moisture content determination; the determination of ash content adopts the constant weight method of burning, referring to the method in GB 5009.4-2016 [21]; the determination of crude protein content follows AACC Method 46-11A, with a protein conversion factor of 5.7.

## 3. Mathematical Modeling of the Crushing Process

The graded crushing process of the blade crusher mainly involves three mechanical processes: blade–particle collision, radial movement of crushed particles along the blade working surface, and particle—crushing-chamber-wall collision. Wheat grains belong to biomaterials with obvious tissue heterogeneity, and there are differences in composition, structure, and mechanical properties among the bran, endosperm, and embryo. There is also a clear spatial composition gradient within the starchy endosperm [22]. Furthermore, the different tissues and structural states of wheat grains exhibit distinct fracture characteristics [23]. The analytical model established in this article does not further distinguish the local mechanical properties of different tissues, but equivalently treats wheat grains or their broken particles as particles with concentrated mass and equivalent mechanical parameters. Therefore, the following model is mainly used to describe the overall mechanical changes during particle collision and motion, rather than to describe the local fracture behavior of different tissues.

### 3.1. Impact Mechanics Model of Blade–Particle Collision

**Figure 2:**
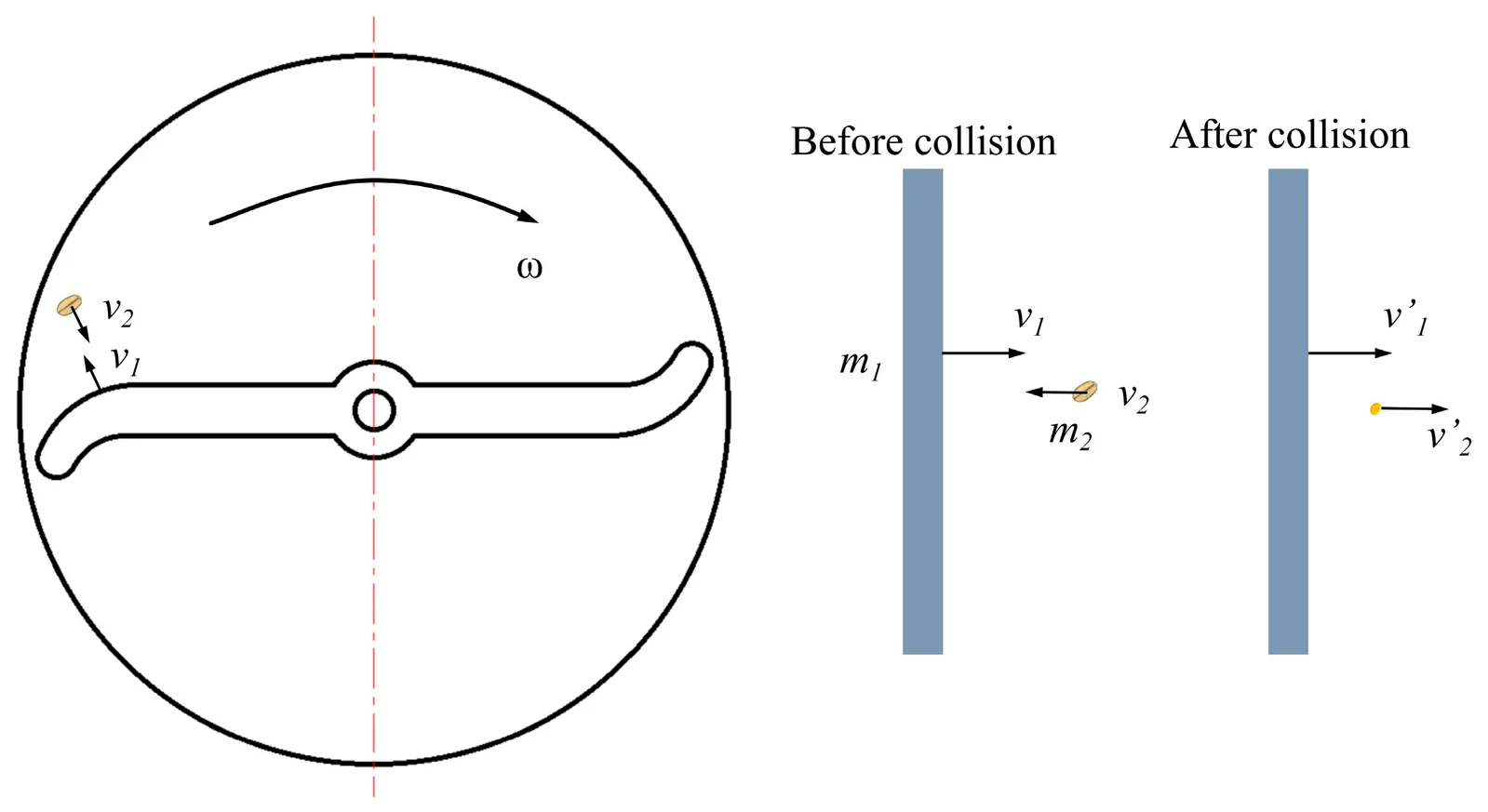
Schematic diagram of wheat grain colliding with blade.

During operation of the blade crusher, wheat kernels and their fragments are repeatedly subjected to impact by the high-speed rotating blades. To obtain an analytically tractable description of the blade–particle interaction, the collision is simplified as a one-dimensional collision along the principal impact direction. Let *m_1_* denote the effective mass of the blade in the impact direction and *m_2_* the mass of the wheat particle. To avoid possible multi fragment fracture behavior during collision, the wheat particles and the fragments generated after collision are treated as an equivalent particle system. The blade velocity after collision is denoted as $v_{1}^{\prime }$, and the equivalent center of mass velocity of the particle system is denoted as $v_{2}^{\prime }$. As shown in Figure 2.

According to the law of conservation of momentum,

(1) $$m_{1}v_{1}+m_{2}v_{2}=m_{1}v_{1}^{\prime }+m_{2}v_{2}^{\prime }$$

The coefficient of restitution e is introduced to describe the relative velocity change during collision:

(2) $$e=\frac{v_{2}^{\prime }-v_{1}^{\prime }}{v_{1}-v_{2}}$$

where 0≤e≤1. The condition e=0 corresponds to a fully inelastic collision and is regarded only as a limiting case rather than as an experimentally verified description of the actual wheat–blade collision. Experimental studies have shown that the coefficient of restitution of wheat kernels varies with impact velocity and moisture content.

Combining Equations (1) and (2) gives

(3) $$v_{1}^{\prime }=\frac{\left(m_{1}-em_{2}\right)v_{2}+\left(1+e\right)m_{2}v_{2}}{m_{1}+m_{2}}$$

(4) $$v_{1}^{\prime }=\frac{\left(m_{2}-em_{1}\right)v_{2}+\left(1+e\right)m_{1}v_{1}}{m_{1}+m_{2}}$$

The momentum transmitted from the blade to the wheat grains is

(5) $$P=m_{2}\left(v_{2}^{\prime }-v_{2}\right)$$

Substitution of Equation (4) into Equation (5) yields

(6) $$P=\frac{\left(1+e\right)m_{1}m_{2}}{m_{1}+m_{2}}\left(v_{1}-v_{2}\right)$$

If ∆t denotes the effective contact duration, the average impact force is

(7) $$F=\frac{P}{∆t}$$

Therefore,

(8) $$F=\frac{\left(1+e\right)m_{1}m_{2}}{\left(m_{1}+m_{2}\right)∆t}\left(v_{1}-v_{2}\right)$$

The linear velocity of the blade at the collision radius *R* is

(9) $$v_{1}=\omega R=\frac{\pi n_{1}R}{30}$$

where $n_{1}$ is the rotational speed of the crusher main shaft and *ω* is the angular velocity.

Substitution of Equation (9) into Equation (8) gives

(10) $$F=\frac{\left(1+e\right)m_{1}m_{2}}{\left(m_{1}+m_{2}\right)∆t}\left(\frac{\pi n_{1}R}{30}-v_{2}\right)$$

Equation (10) indicates that, under the simplified collision assumptions and with the other parameters held constant, the average collision force is theoretically related to the relative impact velocity between the blade and the particle. Increasing the blade rotational speed $n_{1}$ or collision radius *R* increases the blade linear velocity and therefore tends to increase the relative impact velocity.

The kinetic energy dissipated during the idealized collision is [24]

(11) $$∆E_{diss}=\frac{1}{2}\;\frac{m_{1}m_{2}}{m_{1}+m_{2}}\left(1-e^{2}\right){\left(v_{1}-v_{2}\right)}^{2}$$

Substituting Equation (9) gives

(12) $$∆E_{diss}=\frac{1}{2}\;\frac{m_{1}m_{2}}{m_{1}+m_{2}}\left(1-e^{2}\right){\left(\frac{\pi n_{1}R}{30}-v_{2}\right)}^{2}$$

When the wheat particle velocity is much smaller than the blade velocity, i.e., $v_{2}\ll v_{1}$, Equation (12) can be approximated as

(13) $$∆E_{diss}\approx \frac{1}{2}\;\frac{m_{1}m_{2}}{m_{1}+m_{2}}\left(1-e^{2}\right){\left(\frac{\pi n_{1}R}{30}\right)}^{2}$$

Thus, the collision-associated kinetic-energy dissipation theoretically increases with the square of the blade linear velocity. However, $∆E_{diss}$ represents the mechanical energy dissipated during the idealized collision and should not be interpreted as the energy consumed exclusively in wheat fragmentation. In an actual crushing process, the dissipated energy may also be associated with particle deformation, friction, heat generation, vibration, rotation, and other energy-loss mechanisms.

Because *e*, *v_2_*, and Δ*t* were not directly measured under the present blade crushing conditions, Equations (10)–(13) are used only to describe theoretical parameter dependencies and not to calculate the actual collision force or crushing energy inside the crusher.

According to the theoretical model, increasing the blade’s rotational speed or collision radius increases the relative impact velocity and consequently increases the average impact force and collision-associated kinetic-energy dissipation. A shorter collision duration increases the average impact force, whereas a higher coefficient of restitution increases the collision impulse but reduces the kinetic-energy dissipation.

### 3.2. Dynamic Analysis of Wheat Grains Sliding Radially Along the Blade Working Surface

After blade impact, some fragmented particles may collide directly with the chamber wall, whereas others may move along the blade working surface before leaving the blade. To describe this motion, axial particle motion is neglected, the blade is treated as a rigid rotating surface, and the fragmented material is represented as a point mass. The initial radial position and radial velocity are denoted by $\rho_{0}$ and $v_{r,0}$ respectively. The tangential velocity associated with blade rotation is

(14) $$v_{\tau }=\omega \rho$$

where *ρ* is the instantaneous radial position of the particle.

**Figure 3:**
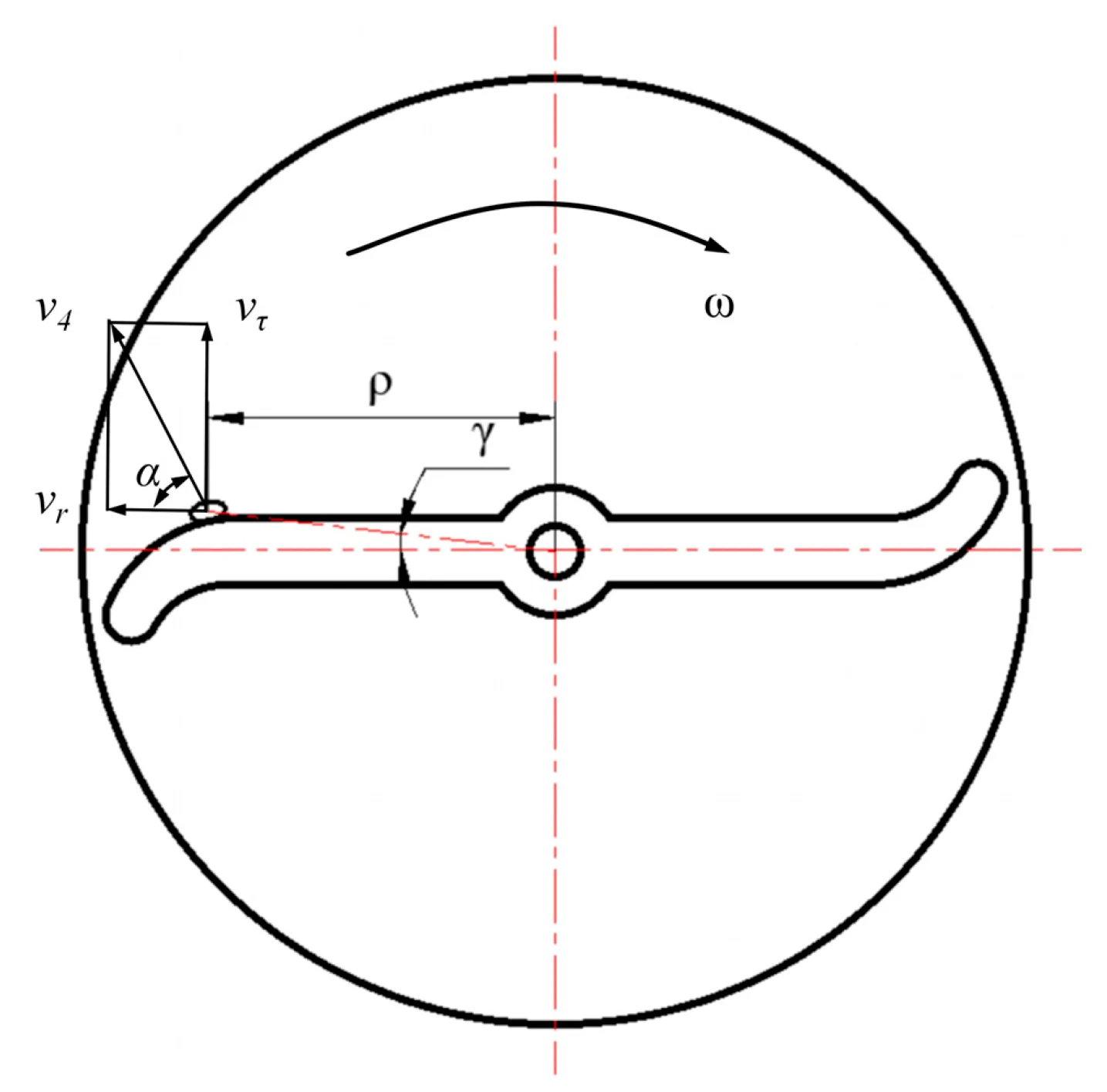
Velocity vector diagram of wheat crushed material along the blade working surface.

The absolute particle velocity ${\vec{v}}_{4}$ is obtained from the vector combination of the radial relative velocity ${\vec{v}}_{r}$ and tangential velocity ${\vec{v}}_{\tau }$. Based on the velocity synthesis theorem [25], a velocity vector diagram is constructed as shown in Figure 3.

(15) $${\vec{v}}_{4}={\vec{v}}_{r}+{\vec{v}}_{\tau }$$

(16) $$v_{4}^{2}=v_{r}^{2}+{\left(\omega \rho \right)}^{2}-2v_{r}\omega \rho \sin\alpha$$

**Figure 4:**
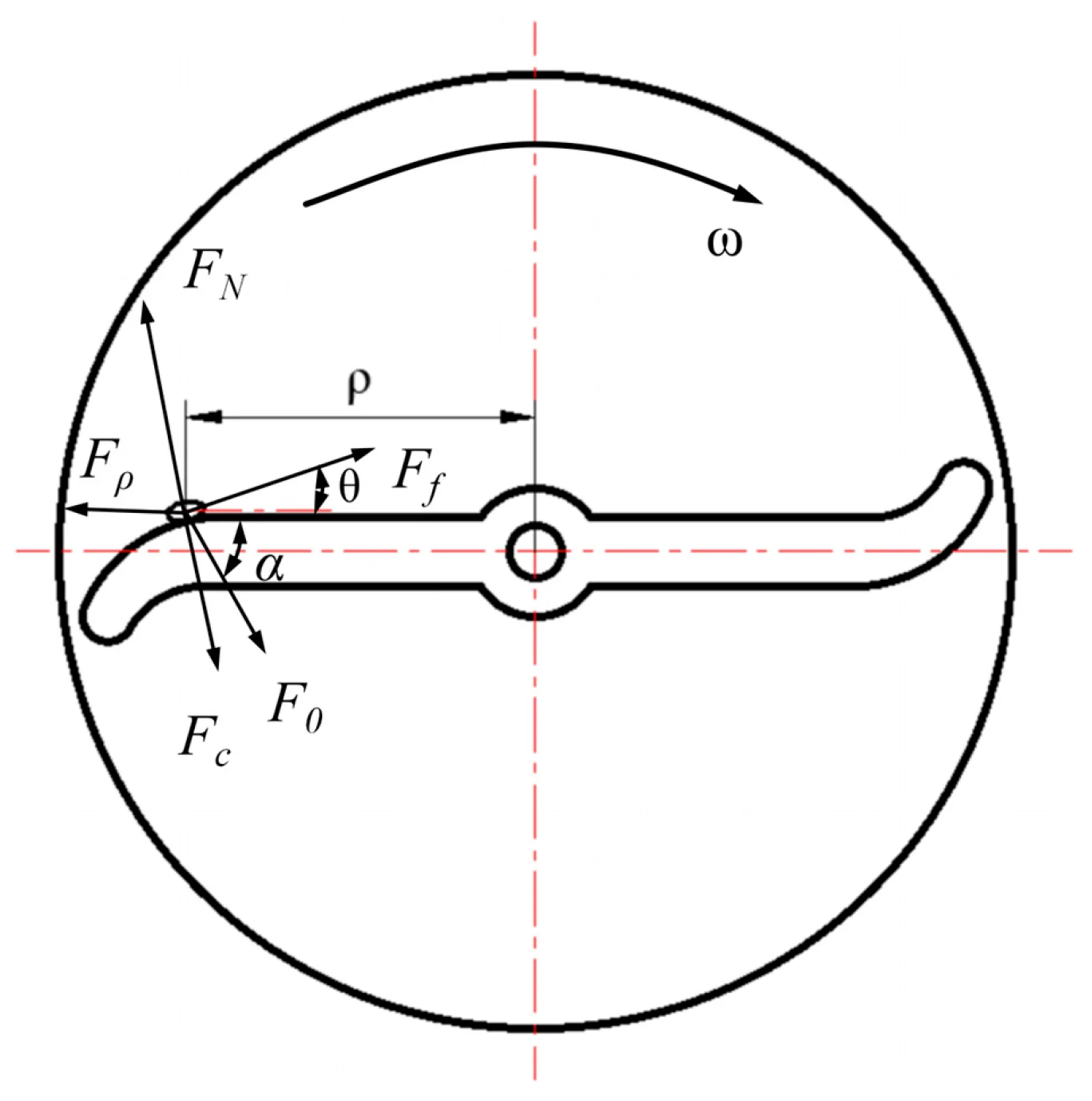
Mechanical model of blade material collision.

As shown in Figure 4, the external forces exerted on the crushed material during the radial sliding process on the blade working surface are mainly Coriolis force *F_c_*, airflow pressure *F_0_*, centrifugal force *F_ρ_*, frictional force *F_f_*, and support force *F_N_*. If the mass of the crushed material is *m_3_*, the magnitude of the force exerted on the material is [26]

(17) $$F_{c}=2m_{3}\omega v_{r}$$

(18) $$F_{\rho }=m_{3}\rho \omega^{2}$$

(19) $$F_{0}=\frac{1}{2}CA\rho_{f}v_{4}^{2}=\frac{1}{2}k\rho_{f}v_{4}^{2}$$

(20) $$F_{f}=\mu F_{N}$$

In the formula, *ω* is the rotational angular velocity of the crusher’s main shaft, rad/s; *ρ* is the rotational radius of the crushed material, m; *C* is the drag coefficient, constant; and *A* is the projected area perpendicular to the airflow direction. For a given crushed material, the projected area remains constant, making this component a constant, m^2^; *k* is the product of drag coefficient *C* and projected area *A*, constant; *ρ_f_* is the airflow density, kg/m^3^; and *µ* is the friction coefficient.

The crushed material is assumed to move along the blade working surface in the radial direction. The radial position of the material element is denoted by *ρ*, and its radial velocity is defined as $v_{r}=d\rho /dt$. Therefore, $dv_{r}/dt=d^{2}\rho /dt^{2}$ represents the radial acceleration of the crushed material.

The differential equation for the motion of crushed materials along the blade working surface is [27]

(21) $$m_{3}\frac{dv_{r}}{dt}=m_{3}\rho \omega^{2}\cos\theta -\frac{1}{2}k\rho_{f}v_{4}^{2}\cos\alpha -\mu F_{N}$$

where the first term on the right-hand side is the tangential component of the centrifugal force, the second term is the tangential component of the aerodynamic resistance, and the third term is the friction force acting along the blade working surface.

For the vertical blade working surface, the force balance equation for material fragmentation is

(22) $$F_{N}+m_{3}\rho \omega^{2}\sin\theta -\frac{1}{2}k\rho_{f}v_{4}^{2}\sin\alpha -2m_{3}\omega v_{r}=0$$

Substituting this expression into Equation (21) yields

(23) $$\frac{dv_{r}}{dt}=\rho \omega^{2}\left(\cos\theta +\mu \sin\theta \right)-\frac{k\rho_{f}v_{4}^{2}}{2m_{3}}\left(\cos\alpha +\mu \sin\alpha \right)-2\mu \omega v_{r}$$

Consequently, the governing equation for the radial motion of the crushed material can be written as

(24) $$\begin{array}{l} \frac{dv_{r}}{dt}=\rho \omega^{2}\left(\cos\theta +\mu \sin\theta \right)-\frac{1}{2m_{3}}k\rho_{f}\left[v_{r}^{2}+{\left(\omega \rho \right)}^{2}-2v_{r}\omega \rho \sin\alpha \right]\\ \left(\cos\alpha +\mu \sin\alpha \right)-2\mu \omega v_{r} \end{array}$$

In Equations (21)–(23), *m_3_* is the mass of the crushed material element; *v_r_* is the radial velocity of the material; and the angle *θ* represents the angle between the centrifugal-force direction and the blade working surface, whereas *α* represents the angle between the relative aerodynamic-force direction and the blade working surface.

Equation (24), together with $v_{r}=d\rho /dt$, forms the governing differential-equation system for the radial motion of the crushed material. *A* quantitative solution of this system requires the initial conditions

(25) $$\rho \left(0\right)=\rho_{0},\;\;\;\;\;\;\;\;v_{r}\left(0\right)=v_{r,0}$$

where *ρ_0_* and *v_r,0_* are the radial position and radial velocity of the material when it first enters the blade working surface.

Thus, Equation (24), together with Equation (25), constitute the governing differential-equation system for radial particle motion. A quantitative solution requires experimentally determined initial conditions and effective parameters, including $v_{r,0}$, μ, C, and A. These parameters were not independently determined in the present experiments. Therefore, the governing equations were not numerically solved and are used only to indicate that particle radial motion is theoretically influenced by rotational speed, radial position, aerodynamic resistance, friction, and initial particle motion.

The kinetic energy of a fragmented particle leaving the blade can be expressed as [28]

(26) $$T_{3}=\frac{1}{2}m_{3}v_{4}^{2}=\frac{1}{2}m_{3}\left[v_{r}^{2}+{\left(\omega \rho \right)}^{2}-2v_{r}\omega \rho \sin\alpha \right]$$

Equation (26) describes the theoretical dependence of particle kinetic energy on its instantaneous radial and tangential velocities. Because these particle velocities were not directly measured, the equation is not used to quantitatively determine the kinetic energy of particles leaving the blade.

The radial motion of particles along the blade surface is jointly governed by rotational speed, radial position, aerodynamic drag, and friction. Increasing the rotational speed or radial position enhances the centrifugal contribution to particle motion, whereas aerodynamic resistance and friction oppose radial sliding.

### 3.3. Collision Mechanics Analysis of the Side Wall of the Crushing Chamber on the Material

**Figure 5:**
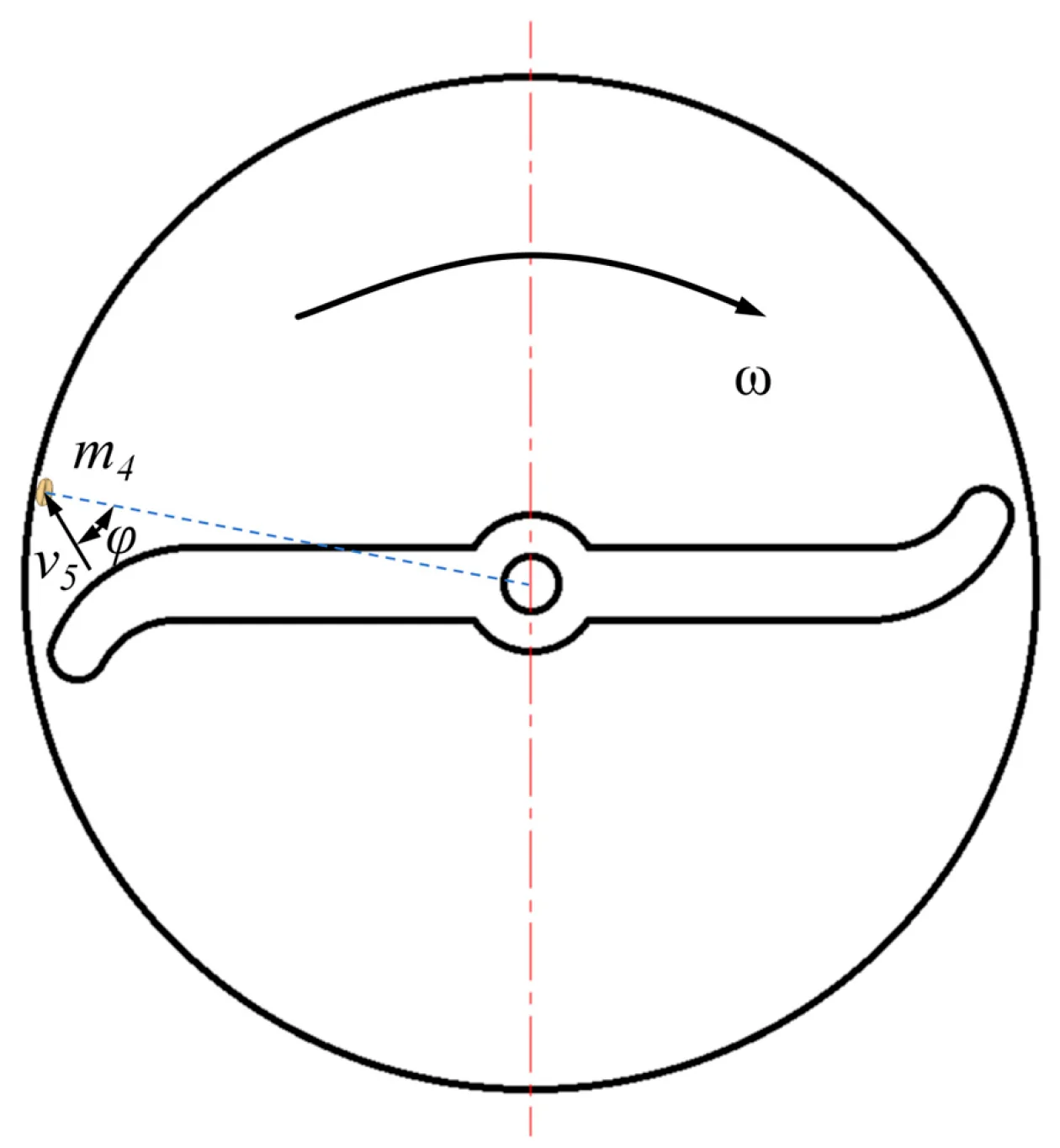
Model of collision velocity between wheat and the side wall of the crushing chamber.

After leaving the blade, fragmented particles may collide with the chamber wall and undergo further mechanical loading. Let $m_{4}$ denote the particle mass, $v_{5}$ its velocity immediately before wall impact, and *φ* the incidence angle measured from the wall normal, as shown in Figure 5. The normal and tangential components of the impact velocity are

(27) $$v_{5,n}=v_{5}\cos\varphi$$

(28) $$v_{5,t}=v_{5}\sin\varphi$$

In order to consider the rebound during the collision process, if the coefficient of restitution during the collision between the particle–wall is $e_{w}$, then

(29) $$e_{w}=-\frac{v_{5,n}^{\prime }}{v_{5,n}}$$

where $v_{5,n}^{\prime }$ is the post-impact normal velocity component.

For an idealized collision with a rigid chamber wall, the normal impulse is

(30) $$P_{w}=m_{4}\left(1+e_{w}\right)v_{5}\cos\varphi$$

If $∆t_{1}$ is the effective wall-contact duration, the average normal collision force is

(31) $$F_{1}=\frac{m_{4}\left(1+e_{w}\right)v_{5}\cos\varphi }{∆t_{1}}$$

The kinetic energy dissipated in the normal direction is

(32) $$∆E_{n}=\frac{1}{2}m_{4}\left(1-e_{w}^{2}\right)v_{5}^{2}\cos^{2}\varphi$$

For tangential sliding during wall contact, the frictional force can be approximately described as

(33) $$F_{t}=\mu_{w}F_{N}$$

The work done by frictional force is

(34) $$W_{f}=\int F_{t}ds$$

where $\mu_{w}$ is the effective particle–wall friction coefficient and *s* is the tangential sliding distance.

The total mechanical energy dissipated during the simplified particle–wall interaction can therefore be expressed as

(35) $$∆E_{w}=∆E_{n}+W_{f}$$

According to Equation (31), when the other parameters remain constant, decreasing φ increases the normal component of the collision impulse and the corresponding average normal collision force. Equation (32) likewise indicates that the normal component of collision-associated energy dissipation depends on the incidence angle. These relationships provide a qualitative mechanical explanation for the possible contribution of particle–wall collisions to continued particle fragmentation.

## 4. Result and Discussion

### 4.1. Distribution of Wheat Particles in Graded Crushing Process

**Figure 6:**
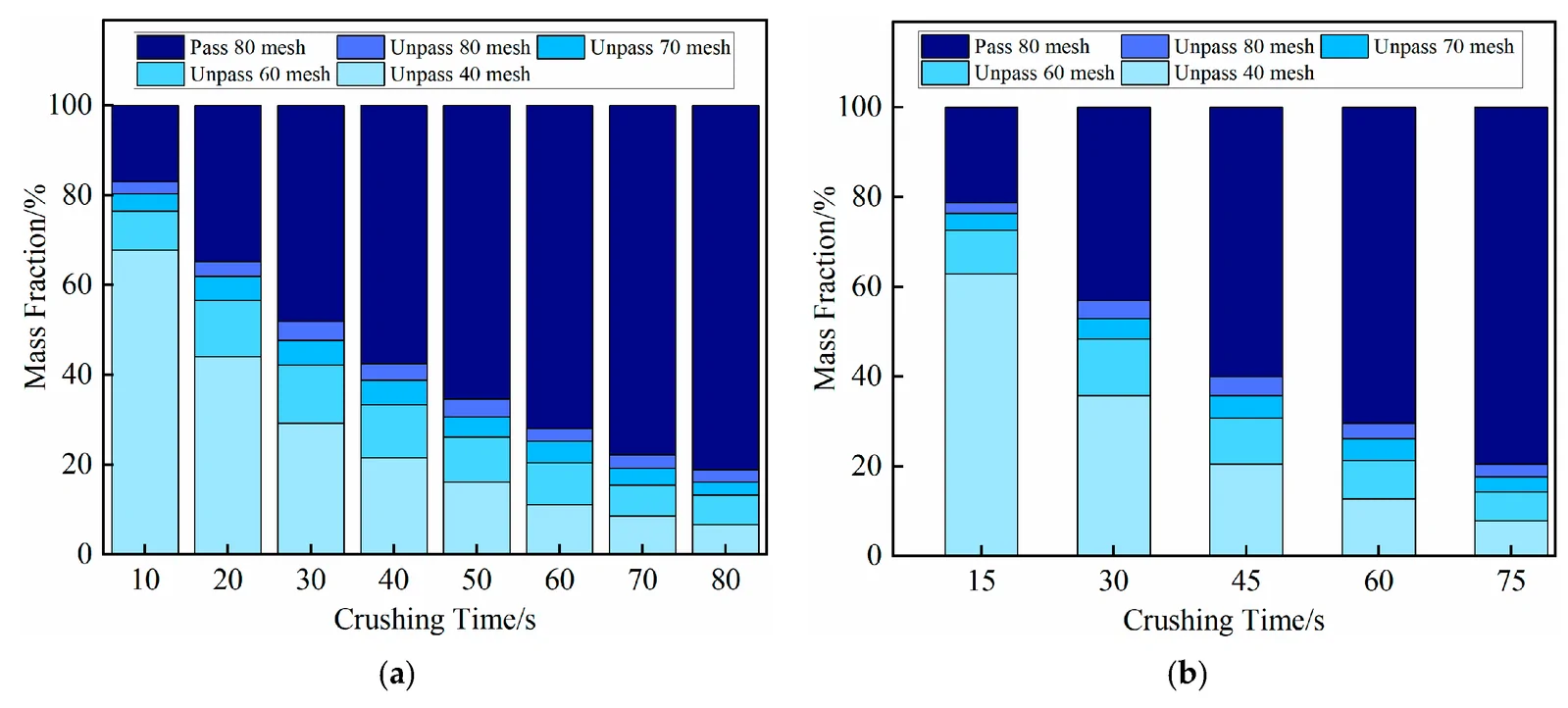
Evolution of particle size distribution during the crushing process of graded crushed wheat. (**a**) 10 s graded crushing process; (**b**) 15 s graded crushing process.

Figure 6a,b illustrate the evolution of wheat particle size distribution during the graded crushing process at 10 and 15 s per pass, respectively. Results show that both processes exhibit a common trend: the mass fraction of large particles (>40 mesh) continuously decreases, while that of small particles (<80 mesh) gradually increases. Concurrently, the mass fraction of intermediate-sized particles (60~80 mesh) follows a pattern of an initial increase followed by a decrease. This indicates a gradual transformation of particles from coarse to fine during crushing. Notably, in the 15 s graded crushing process, the proportion of large particles decreases more rapidly in the initial stage (30 s). As the crushing process progresses, the rates of change in the mass fractions of all particle-size ranges gradually decrease, indicating the system gradually enters a dynamic equilibrium phase. This may be related to the continuous reduction in the total amount of endosperm material available for crushing. A similar trend has also been reported for corn crushing. As crushing time increases, the content of large particles gradually decreases while the content of small particles significantly increases [29]. This further suggests that the blade crushing process involves crushing large particles into intermediate particles, followed by crushing intermediate particles into small particles. However, these fragmentation processes may occur simultaneously. As crushing time increases, the process gradually enters a dynamic equilibrium phase. Once equilibrium is reached, continued crushing no longer alters particle size [30].

**Figure 7:**
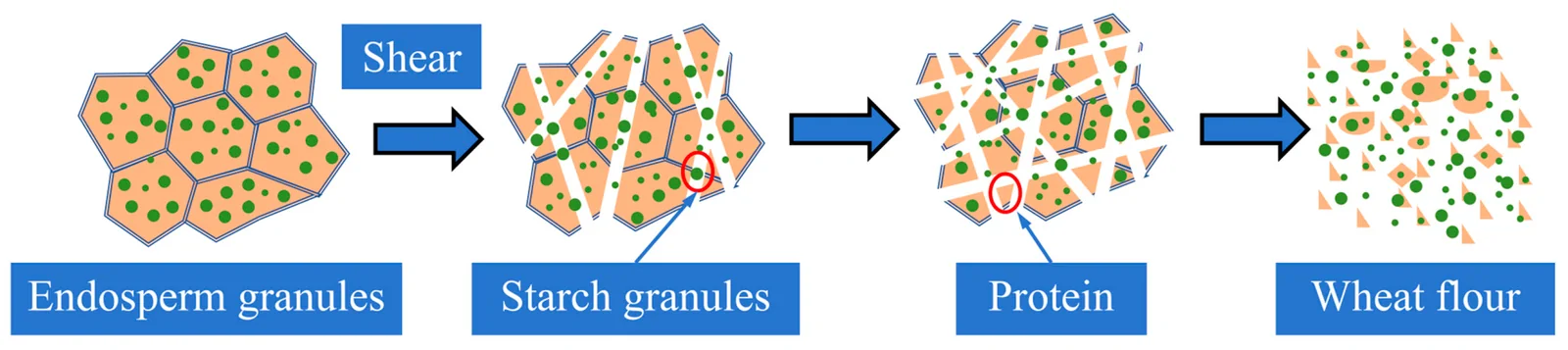
Mechanism diagram of wheat crushing.

Based on the evolution patterns of wheat particle size distribution, and combined with theoretical analysis of crushing models, the crushing process of wheat particles in blade crushers primarily manifests as a “volume crushing” mechanism. This mechanism refers to the overall fracture of macroscopic endosperm clusters under mechanical force—which are gradually broken into small endosperm particles with significantly reduced particle size—and at the same time release smaller starch particles and protein fragments inside. This process not only leads to an overall decrease in particle size but also causes significant changes in powder composition and morphology [31,32], as shown in Figure 7.

### 4.2. Distribution Characteristics of Quantity Particle Size of Graded Crushed Wheat Flour

**Figure 8:**
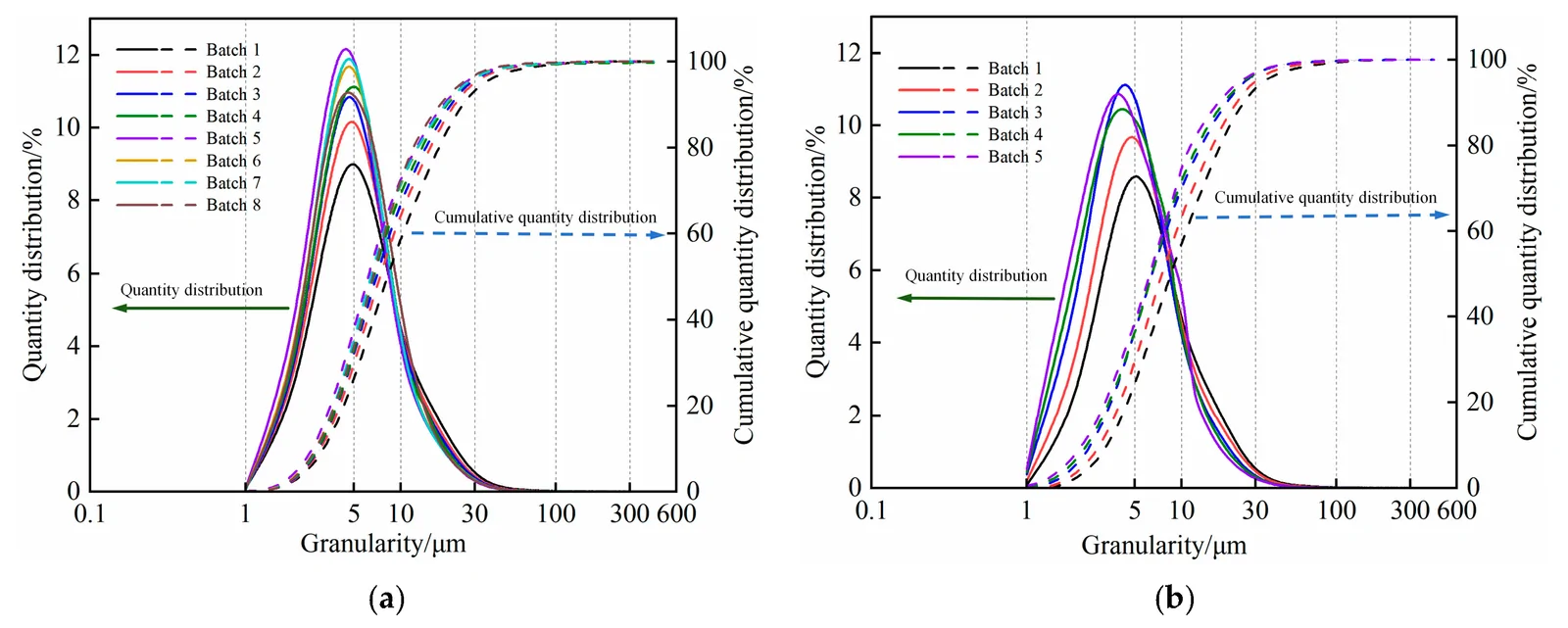
Quantity particle size distribution curve of wheat flour for each pass of graded crushing. (**a**) Using F10 grading crushing technology; (**b**) using F15 grading crushing technology. The solid lines represent the quantity distribution curves of particles, while the dashed lines represent the corresponding cumulative quantity distribution curves. Curves with the same color correspond to the same batch.

Particle size, defined as a measure of particle diameter or dimensions, serves as one of the key indicators for evaluating the processing precision of wheat flour [33,34]. Particle size distribution generally refers to the proportion of endosperm particles within a specific size range relative to the entire wheat flour system. In practical testing, this proportional relationship is commonly quantified using either interval distribution or cumulative distribution, both of which represent important quality parameters for characterizing flour particle properties [35]. Figure 8a shows the quantity particle size distribution curves of wheat flour obtained from each batch during the F10 graded crushing process. The results indicate that the distribution curves for different batches exhibit similar shapes, all concentrated within the 1~100 μm range and displaying a distinct unimodal distribution. The peak particle size remained stable at approximately 5 μm, suggesting a concentrated and uniform particle size distribution under this crushing condition. As the number of crushing passes increased, the peak height of the distribution curve gradually increased and eventually stabilized. This trend is consistent with reports from the existing literature on modern wheat crushing processes, where the refinement of wheat is not directly achieved through a single crushing process, but rather gradually approaches the target particle size distribution through multiple stages of crushing and intermediate screening [36]. During this process, the screening system can separate fine particles that have reached the target size in a timely manner, thereby avoiding excessive crushing caused by continuous mechanical treatment and helping to improve the uniformity of the particle size distribution [22]. Figure 8b presents the corresponding distribution curves for the F15 technology. Despite being collected from different crushing stages, the curves were highly consistent in shape, all showing a single dominant peak within 1~100 μm. As the crushing process progressed, the peak intensity significantly increased, while the peak shape gradually narrowed. Comparison of the particle size distribution curves of the F10 and F15 processes showed that the peak particle size of the powder obtained from F15 was less than 5 μm, and the distribution curve shifted toward smaller particle sizes, indicating a higher relative number frequency of small particles. This further indicates that crushing time has a significant effect on the particle size distribution of wheat flour. The fundamental reason for the differences in particle size distribution and flour composition during the graded crushing stage is the significant spatial heterogeneity of wheat endosperm in terms of tissue structure and chemical composition. Previous studies have demonstrated a clear radial gradient in protein distribution, with protein concentration being higher in the outer starchy endosperm and decreasing toward the central region of the kernel [37]. Starch content and starch-granule populations also vary spatially across the endosperm, with differences in the relative proportions of large A-type and small B-type starch granules among different tissue regions [38]. These compositional and structural gradients are closely associated with the milling behavior and functional properties of wheat flour [39]. Quantitative analyses of radial sections and sequentially fractionated wheat kernels have further confirmed the increase in protein concentration from the inner to the outer endosperm [40]. Based on these observations, the compositional differences among flour fractions obtained at different crushing stages may be partly associated with the differential release and mixing of material originating from compositionally distinct regions of the endosperm during crushing.

The characteristic particle size data of wheat flour are shown in Table 2, where Span is an important indicator of the width of the particle size distribution. A smaller Span value indicates a narrower and more concentrated particle size distribution. From the evolution of the characteristic particle sizes D10, D50, and D90, it can be seen that although the characteristic particle size has decreased, the magnitude is relatively small, indicating that the graded crushing technology effectively avoids excessive crushing while achieving wheat grading refinement, and that the particle size transition between each pass is smooth. The Span value of the particle size distribution concentration index is maintained in the range of 2.46~2.68, and the particle size distribution of the powder is always relatively uniform and concentrated. This indicates that the graded crushing technology achieves stable refinement of particles through step-by-step and controllable mechanical action, avoids local excessive crushing, and ensures the continuity and consistency of powder particle size in each stage, reflecting the excellent performance of this process in particle size control.

### 4.3. The Effect of Graded Crushing on the Content of Damaged Starch in Wheat Flour

**Figure 9:**
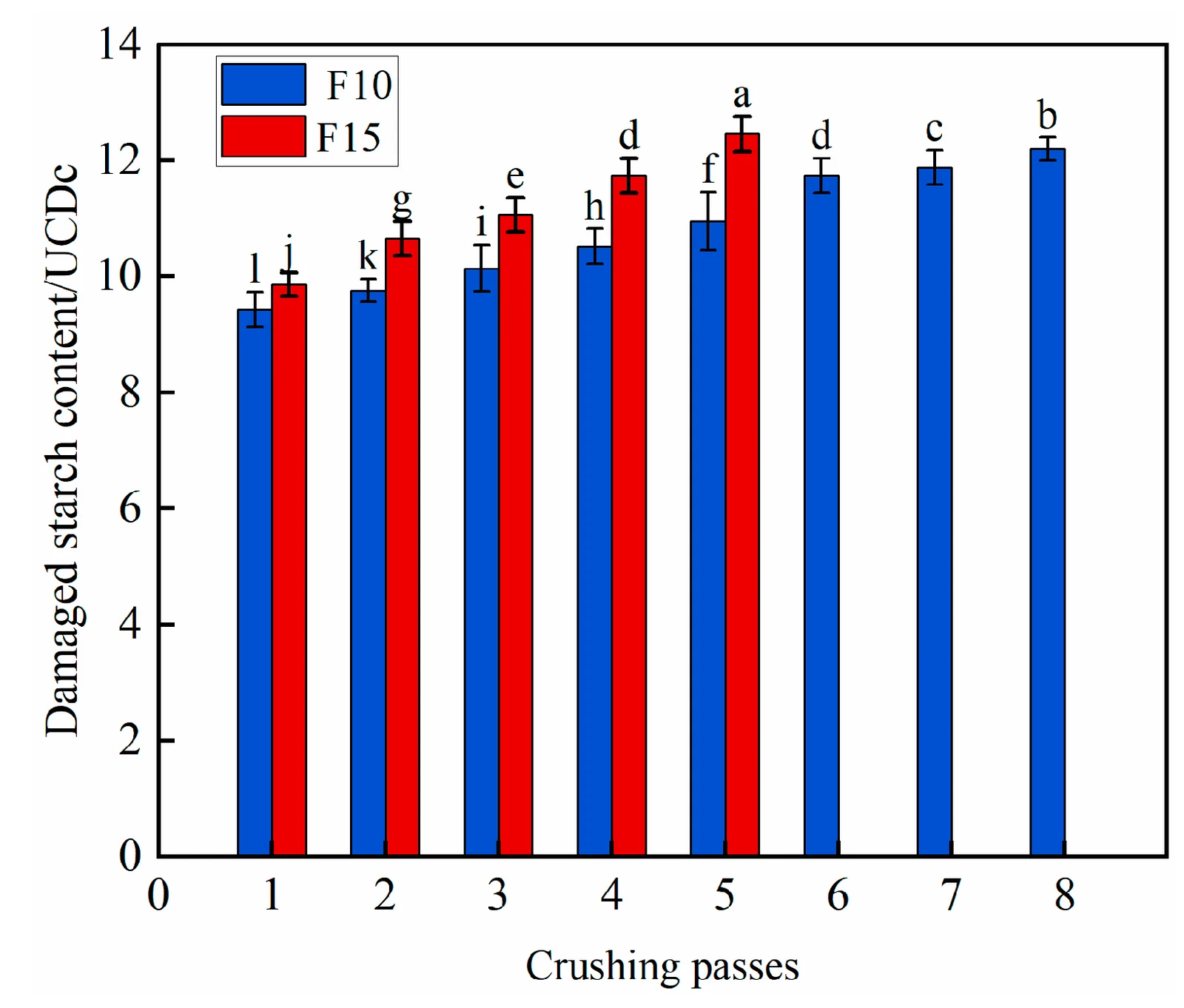
The effect of graded crushing process on the content of damaged starch in wheat flour. Note: Different lowercase letters on the bar chart indicate significant differences (*p* < 0.05).

As shown in Figure 9, the content of damaged starch significantly increased with the increase in crushing passes. This phenomenon may be related to the cumulative mechanical effects of impact and collision experienced by wheat particles during continuous crushing, which increase the degree of mechanical damage to starch particles. The damaged starch content of wheat flour obtained by the F10 process was lower than that of the F15 process, suggesting that longer mechanical action time during the F15 crushing process may lead to more starch damage. However, this study did not directly measure the impact strength, collision frequency, and mechanical damage process of individual starch granules; therefore, this explanation should be regarded as a possible mechanism rather than a direct verification of the causal relationships. And when using multi roll experimental grinding to study the characteristics of wheat powder, it was found that with the increase in grinding intensity, the particle size of the powder continued to decrease and the degree of starch damage also increased [41]. Therefore, in the flour milling process, it is of great significance to reasonably control the number of grinding passes and the strength of action in order to control the content of damaged starch and ensure the quality of the flour. Previous studies have also shown that damaged starch can substantially influence the processing properties of flour and the quality of end products because its structural and functional characteristics differ from those of native starch [42]. During the hydration process of dough, damaged starch particles have significantly enhanced water absorption capacity due to the formation of surface cracks and internal cavities. Their water absorption can reach 200% to 430% of their own weight, which is much higher than the 39% to 87% observed for starch particles. This effectively improves the water absorption rate of flour and accelerates the hydration process of dough [43]. At the same time, damaged starch is more prone to spontaneous expansion and gelatinization in cold water, promoting the dissolution of linear starch and some branched starch, thereby changing the dispersion state of starch particles and reducing peak viscosity and retrogradation value during heating gelatinization process, which directly affects the gelatinization characteristics and storage stability of flour [44]. However, when the content of damaged starch is too high, its excessive water absorption behavior will lead to an increase in dough viscosity and weaken the continuity and integrity of the gluten network due to competition with gluten protein for limited water, thereby reducing the extensibility and gas holding capacity of the dough and ultimately resulting in a decrease in fermentation height, a decrease in specific volume, and a tendency for the internal crumb structure to become denser [45]. Therefore, in actual production, the damaged starch content should be balanced against dough processing performance, fermentation characteristics, and final product quality, while exceeding this range will have adverse effects on food processing performance.

### 4.4. The Effect of Graded Crushing on the Basic Physicochemical Properties of Wheat Flour

As shown in Table 3, with the increase in the number of crushing passes, the moisture content of wheat flour obtained by both the F10 and F15 fractionated crushing processes exhibited a gradual decreasing trend. This is mainly due to the repeated mechanical impact and friction effects on the material during continuous crushing, which led to an increase in local temperature and thus promoted the evaporation of free water. Moreover, relevant studies have indicated that milling time, grinding method, and particle size all affect the final moisture content of flour; the finer the particle size, the more pronounced the moisture loss tends to be [46].

The protein content showed an initial increase followed by a decrease under both processes, reaching its peak at the 4th–5th pass for the F10 process and at the second pass for the F15 process. This observed trend may be related to the radial heterogeneity in the chemical composition of wheat kernels and the differential contribution of materials from different endosperm regions during successive crushing passes [38]. The distribution of protein within the wheat endosperm is markedly uneven, increasing from the center toward the outer layers, with protein content near the aleurone layer generally higher than that in the central endosperm. Similar findings have also been observed in roller milling: protein content gradually increases as the break and reduction passages proceed, with the highest protein content found in the last break flour stream, while the lowest appears in the early reduction streams derived from the central endosperm region, reflecting a gradient distribution of protein from the inner to the outer endosperm [47].

Ash content exhibited a general upward trend, indicating that as the number of milling passes increased, the endosperm near the outer layers of the kernel, the aleurone layer, and small amounts of bran tissue gradually entered the flour stream. Ash content primarily originates from minerals, which are concentrated in the peripheral tissues of the wheat kernel. Ash content is commonly regarded as an important indicator for evaluating flour extraction rate and degree of refinement; its increase reflects the entry of more mineral-rich outer tissues into the flour [48]. Although a higher ash content contributes to improving the mineral nutritional value of flour, it simultaneously reduces flour whiteness and may adversely affect the processing quality of certain refined flour-based products. Therefore, industrial milling usually requires a trade-off between nutrient retention and product quality. From the perspective of the significance analysis, the trends of major quality indicators under the two processes were generally consistent, and most indicators across different passes showed significant differences (*p* < 0.05), indicating that fractionated grinding has a marked effect on the chemical composition of wheat flour.

### 4.5. Relationship Between Mechanical Models and Experimental Observations

Equations (6) and (10) indicate that, under the assumptions adopted in the theoretical analysis, the impact force and crushing energy increase with blade rotational speed and rotational radius. During graded crushing, as the number of crushing passes increases, the material repeatedly experiences impacts, and the cumulative mechanical energy transferred to the material is expected to increase. The particle size evolution shown in Figure 6 and Figure 8 indicates that the mass fraction of coarse particles continues to decrease, while the intermediate particles show a trend of first increasing and then decreasing. The fine particles gradually increase, exhibiting a typical step-by-step crushing characteristic of “coarse particles → medium particles → fine particles”. The observed behavior suggests that particles in different size ranges may require different levels of impact loading and energy input to undergo further fragmentation.

In addition, the F15 process has a longer single crushing time compared to the F10 process. A longer single-pass crushing duration is expected to increase the number of particle–blade, particle–particle, and particle–chamber interactions, and may result in greater cumulative mechanical energy transfer to the material. Consistent with this interpretation, the particle size distribution obtained using the F15 process shifts towards smaller particle sizes, with a decrease in peak particle size and an increase in the proportion of fine particles. At the same time, the content of damaged starch significantly increases with the increase in crushing passes, and the F15 process is higher than the F10 process. This result is qualitatively consistent with the interpretation that prolonged mechanical action and repeated impact loading increase the extent of mechanical damage to starch granules. The experimental trends are qualitatively consistent with the energy-driven fragmentation mechanism suggested by the theoretical models, but the proposed models are based on simplified assumptions (e.g., ideal geometry, homogeneous properties, and neglect of inter-particle interactions) and thus only offer a qualitative trend interpretation rather than an accurate quantitative prediction of particle size distribution or damaged starch content.

## 5. Conclusions

During the grading and crushing process of the blade crusher, wheat particles exhibit a crushing characteristic of gradually transforming from a coarse grain size to an intermediate and a fine grain size. Compared with the F10 process, the F15 process further promotes particle refinement, causing the overall particle size distribution of the obtained wheat flour to shift towards smaller particle sizes. With the increase in crushing time, the content of damaged starch significantly increases, and the F15 process is generally higher than the F10 process, indicating that the longer single-pass mechanical action not only promotes particle refinement, but also exacerbates the mechanical damage of starch particles.

Graded crushing simultaneously causes systematic changes in the physicochemical composition of wheat flour. Under both processes, the moisture content gradually decreases with the crushing process, while the protein content shows a trend of first increasing and then decreasing. The ash content continues to increase and eventually reaches about 0.48%. From this, it can be seen that the duration and number of crushing passes not only affect the gradual refinement process of particles, but also accompany changes in starch damage and physicochemical composition.

The blade–particle collision, radial sliding of particles along the blade surface, and particle fragmentation–chamber-wall collision analysis models established in this study are used for the qualitative mechanical interpretation of the observed particle fragmentation trend in experiments, but have not been quantitatively validated through experiments. Future research can further combine discrete element simulation and graded crushing experiments to characterize the motion trajectory, collision frequency, collision speed, and energy distribution of particles inside the blade crusher, and further validate the model through the evolution of particle size and changes in damaged starch under different operating conditions.

## Figures and Tables

**Table 1:** Relevant equipment parameters.

Equipment Category	Core Parameters	Parameter Value
Blade mill	Rated Power	2.1 kW
	Operating speed	32,000 r/min
	Frequency	50/60 Hz
	Crushing chamber diameter	160 mm
Vibrating screen	Excitation power	0.18 kW
	Frequency	50/60 Hz
	Operating speed	1500 r/min
	Screening time	5 min

**Table 2:** Characteristic particle size data for graded crushed wheat flour.

Sample Name	D10 (μm)	D50 (μm)	D90 (μm)	Span
F10-1 batch	3.27 ± 0.27 ^ab^	8.36 ± 0.47 ^a^	25.47 ± 0.77 ^a^	2.66 ± 0.08 ^d^
F10-2 batch	3.06 ± 0.11 ^abc^	7.38 ± 0.17 ^b^	22.33 ± 0.32 ^bc^	2.61 ± 0.03 ^d^
F10-3 batch	3.01 ± 0.23 ^abc^	7.07 ± 0.43 ^b^	21.33 ± 0.11 ^c^	2.59 ± 0.03 ^d^
F10-4 batch	2.89 ± 0.76 ^d^	6.75 ± 0.69 ^de^	19.94 ± 0.32 ^ef^	2.52 ± 0.26 ^c^
F10-5 batch	2.68 ± 0.06 ^c^	6.17 ± 0.11 ^cd^	18.90 ± 0.14 ^def^	2.63 ± 0.03 ^d^
F10-6 batch	2.83 ± 0.12 ^bc^	6.48 ± 0.19 ^c^	19.52 ± 0.51 ^de^	2.57 ± 0.02 ^d^
F10-7 batch	2.79 ± 0.02 ^c^	6.29 ± 0.09 ^c^	19.02 ± 0.31 ^def^	2.58 ± 0.01 ^d^
F10-8 batch	2.84 ± 0.11 ^e^	6.52 ± 0.34 ^f^	19.06 ± 0.56 ^g^	2.48 ± 0.05 ^ab^
F15-1 batch	3.32 ± 0.11 ^a^	8.57 ± 0.21 ^a^	25.27 ± 0.37 ^a^	2.56 ± 0.03 ^d^
F15-2 batch	2.97 ± 0.05 ^abc^	7.50 ± 0.11 ^b^	22.73 ± 0.33 ^b^	2.63 ± 0.02 ^d^
F15-3 batch	2.63 ± 0.17 ^c^	6.53 ± 0.29 ^c^	19.75 ± 0.65 ^d^	2.62 ± 0.04 ^d^
F15-4 batch	2.43 ± 0.09 ^e^	6.43 ± 0.17 ^e^	18.79 ± 0.46 ^f^	2.54 ± 0.03 ^b^
F15-5 batch	2.28 ± 0.05 ^e^	6.39 ± 0.15 ^f^	17.97 ± 0.31 ^g^	2.46 ± 0.04 ^a^

Note: The results are expressed as average value ± standard deviation, and different lowercase letters in the same column indicate significant differences (*p* < 0.05) (the same below).

**Table 3:** Physical and chemical characteristics of wheat flour in each pass of graded crushing process.

Sample Name	Moisture Mass Fraction/%	Protein Mass Fraction/%	Ash Mass Fraction/%
F10-1 batch	14.30 ± 0.07 ^a^	11.72 ± 0.03 ^ef^	0.42 ± 0.01 ^de^
F10-2 batch	14.08 ± 0.06 ^b^	11.91 ± 0.07 ^c^	0.42 ± 0.01 ^de^
F10-3 batch	13.85 ± 0.10 ^c^	12.08 ± 0.03 ^b^	0.43 ± 0.01 ^de^
F10-4 batch	13.66 ± 0.11 ^de^	12.21 ± 0.07 ^a^	0.43 ± 0.01 ^d^
F10-5 batch	13.52 ± 0.10 ^ef^	12.25 ± 0.05 ^a^	0.45 ± 0.02 ^c^
F10-6 batch	13.22 ± 0.09 ^g^	11.85 ± 0.08 ^cd^	0.46 ± 0.01 ^bc^
F10-7 batch	13.10 ± 0.07 ^g^	11.82 ± 0.05 ^cd^	0.48 ± 0.01 ^a^
F10-8 batch	12.86 ± 0.09 ^h^	11.63 ± 0.07 ^f^	0.48 ± 0.01 ^a^
F15-1 batch	14.25 ± 0.08 ^a^	11.90 ± 0.07 ^c^	0.41 ± 0.01 ^e^
F15-2 batch	13.75 ± 0.11 ^cd^	12.24 ± 0.05 ^a^	0.43 ± 0.01 ^de^
F15-3 batch	13.46 ± 0.12 ^f^	12.02 ± 0.04 ^b^	0.46 ± 0.01 ^bc^
F15-4 batch	13.16 ± 0.11 ^g^	11.78 ± 0.07 ^de^	0.47 ± 0.01 ^ab^
F15-5 batch	12.73 ± 0.10 ^h^	11.63 ± 0.02 ^f^	0.48 ± 0.01 ^a^

Note: Different lowercase letters in the same column indicate significant differences (*p* < 0.05).

## Data Availability

The original contributions presented in the study are included in the article, further inquiries can be directed to the corresponding author.
